# Value of blood inflammatory markers for predicting intravenous immunoglobulin resistance in Kawasaki disease: A systematic review and meta-analysis

**DOI:** 10.3389/fped.2022.969502

**Published:** 2022-08-23

**Authors:** Chang Liu, Jiacheng Wu

**Affiliations:** ^1^Department of Pediatrics, The First Affiliated Hospital of China Medical University, Shenyang, China; ^2^Department of Urology, Affiliated Tumor Hospital of Nantong University & Nantong Tumor Hospital, Nantong, China

**Keywords:** C-reactive protein, intravenous immunoglobulin resistance, Kawasaki disease, meta-analysis, neutrophil-lymphocyte ratio

## Abstract

**Background:**

Previous studies have assessed the diagnostic accuracy of blood inflammatory markers like neutrophil-to-lymphocyte ratio (NLR), platelet-to-lymphocyte ratio (PLR), and C-reactive protein (CRP), and CRP to albumin ratio (CAR) to predict the resistant Kawasaki disease (KD). The aim of the current meta-analysis and systematic review is to compare the prognostic ability of these inflammatory markers to predict the resistance to IVIG in patients with Kawasaki disease.

**Methods:**

A systematic search of online academic databases and search engines such as EMBASE, PubMed Central, MEDLINE, Cochrane library, Google Scholar, and ScienceDirect was conducted for papers that report the diagnostic accuracy of inflammatory markers for resistant KD. Meta-analysis was performed using STATA software.

**Results:**

Twenty-two studies met the inclusion criteria. Pooled sensitivity and specificity of NLR as a predictor of resistant Kawasaki disease was 72% (95% CI: 62%, 80%) and 71% (95% CI: 63%, 78%), with AUC of 0.77 for PLR was 60% (95% CI: 50%, 69%) and 68% (95% CI: 61%, 75%), with area under the curve (AUC) of 0.69. Pooled sensitivity and specificity of CRP was 75% (95% CI: 68%, 81%) and 66% (95% CI: 55%, 76%), respectively, with an AUC value of 0.78. Pooled sensitivity and specificity of combined NLR and PLR was 58% (95% CI: 46%, 69%) and 73% (95% CI: 65%, 79%), respectively, with an AUC value of 0.72.

**Conclusion:**

Our study found that NLR, CRP, PLR, and combined NLR/PLR have a good prognostic value in patients with resistant Kawasaki disease, with moderate to high sensitivity and specificity. More research on the accuracy of these indexes in multiple combinations is needed.

**Systematic review registration:**

[https://www.crd.york.ac.uk/prospero/], identifier [CRD42022322165].

## Introduction

Kawasaki disease (KD) is an acute vasculitis that affects coronary arteries in children (1) and is considered the most common cause of acquired cardiac complications in children worldwide.

Intravenous immunoglobulin (IVIG) is the main method of KD treatment that has been known to reduce the risk of coronary artery lesions to 4% from 25% if initiated on time (2). However, 10–20 percent of the patients with Kawasaki disease are IVIG-resistant and have a recurrent or persistent fever even after the initial treatment with IVIG (2). Studies show that IVIG-resistant patients have higher rates of coronary artery lesions development compared to patients that respond to IVIG therapy (3, 4). Combining IVIG with other anti-inflammatory therapeutic agents as the first treatment might lower the incidence of resistance to IVIG and the number of coronary artery lesions (5–8). It is necessary, therefore, to develop ways of early identification of the patients at higher risk of resistant KD, to allow them to benefit from the rapid and aggressive line of management.

Recently, several studies focused on the ability of various blood inflammatory markers like platelet-to-lymphocyte ratio (PLR), neutrophil-to-lymphocyte ratio (NLR), and C-reactive protein (CRP), and CRP to albumin ratio (CAR) to predict the resistant Kawasaki disease (9–15). There is a need for determination of markers for outcome, including IVIG resistance and coronary artery involvement. There is comprehensive review on possible biomarkers for KD and studies comparing the available ten IVIG resistance-predicting scoring system (16, 17). However, these studies show an unsatisfying sensitivity for predicting IVIG resistance. In addition, there are still no reviews that attempted to summarize this evidence systematically. Hence, the main aim of the current systematic review and meta-analysis is to compare the prognostic values of NLR, PLR, CRP, and CAR for predicting the resistance to IVIG in patients with Kawasaki disease.

## Methods

### Eligibility criteria

#### Study design

Prospective and retrospective observational studies (cohort, case-control, or cross-sectional analytical) were included. Case reports, series, conference abstracts, and unpublished gray literature were excluded. The study was registered on PROSPERO (No. CRD42022322165).

#### Study participants

Studies of the Kawasaki disease patients were incorporated irrespective of age and gender of the participants, and comorbidities.

#### Index test and reference standards

Studies assessing the accuracy of NLR, PLR, CRP, and PAR in predicting the resistant form of Kawasaki disease were included if they satisfied all other inclusion criteria.

### Search strategy

An extensive systematic search was done in the databases and search engines such as PubMed Central, EMBASE, MEDLINE, Cochrane library, Google Scholar, and ScienceDirect. The following combination of medical subject headings (MeSH) and free-text headings with the suitable Boolean operators (“AND” and “OR” and “NOT”) in between the pre-defined search terms was used: (“Mucocutaneous Lymph Node Syndrome”[MeSH Terms] OR “Resistant Kawasaki Disease”) OR AND (“immunoglobulins, intravenous”[MeSH Terms] AND “Neutrophil Lymphocyte Ratio”) OR (“Platelet Lymphocyte Ratio”) OR (“CRP Albumin Ratio”) OR (“C-Reactive Protein”) OR (“Neutrophils”[MeSH Terms]) OR “Lymphocytes”[MeSH Terms] OR “Blood Platelets”[MeSH Terms] OR “C-Reactive Protein”[MeSH Terms] OR “Albumins”[MeSH Terms]. The following additional filters were also applied during the process of literature search: time point (January 1954–March 2022), and language (English and Chinese). References from the retrieved studies were also searched to identify any additional relevant articles.

### Steps in study selection

The first step of the study selection process included a screening of the title, keywords, and abstract and was carried out by the two independent reviewers (CL and JW). Full-text studies were then shortlisted independently by two investigators for the second stage of screening based on the eligibility criteria, and studies that met the criteria were selected for further analysis. Disagreements were resolved by discussion. *“Preferred Reporting Items for Systematic Reviews and Meta-Analyses (PRISMA) statement 2020”* was used for reporting this review (18).

### Data extraction procedure

Data was extracted manually by the two investigators (CL and JW) using a pre-defined semi-structured data collection form that was defined at the stage of the protocol itself. The following information was obtained: name of the authors, study title, publication year, year when the study was done, duration of the study, design, study setting, country/region of the study, sample size, outcome assessment tool, and other details, mean age, index test, reference standards, true- and false-positive and negative values for each inflammatory marker. The first author (CL) entered the data, and the second author (JW) verified the correctness of the data entry.

### Risk of bias assessment

The risk of bias was evaluated independently by two authors (CL and JW) using the quality assessment of diagnostic accuracy studies-2 (QUADAS-2) tool (19). The assessed domains were: patient selection, type and interpretation of index test and reference standards, and flow and timing of outcome assessment. Based on these domains, studies received a high, low, or unclear grade.

### Statistical analysis

Statistical analysis was done by STATA *“Midas”* command package. Pooled indices of predictive accuracy, i.e., sensitivity, specificity, diagnostic odds ratio (DOR), likelihood ratio positive (LRP), and negative (LRN) for the predictive accuracy of inflammatory markers were assessed using bivariate meta-analysis. Graphical representation of the diagnostic accuracy indices was done by the forest plot, LR scattergram (clinical value of inflammatory markers), and Fagan plot (post-test probability of a patient having resistant Kawasaki disease). Summary receive operator characteristic curve (sROC showing area under the curve [AUC]) was done. Between-study variability (heterogeneity) was assessed using the χ^2^ test and I^2^ statistic with graphical representation using a bivariate box plot. Publication bias was statistically assessed using Deek’s test and graphically represented by the funnel plot. *P* < 0.05 was statistical significance.

## Results

### Study selection

**FIGURE 1:**
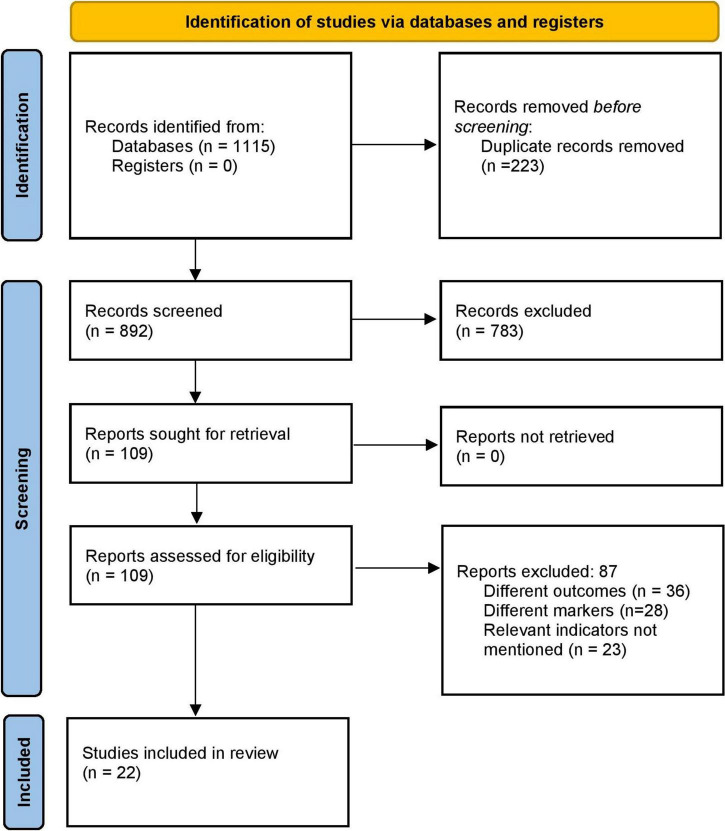
PRISMA flowchart.

The literature search identified 1,115 records. Of them, full texts were obtained from the 109 relevant studies. Three additional articles were identified by screening the references of the retrieved full texts during primary screening. After the final screening against eligibility criteria, 22 papers were included in this study (Figure 1) (13–15, 20–38).

### Characteristics of the included studies

**FIGURE 2:**
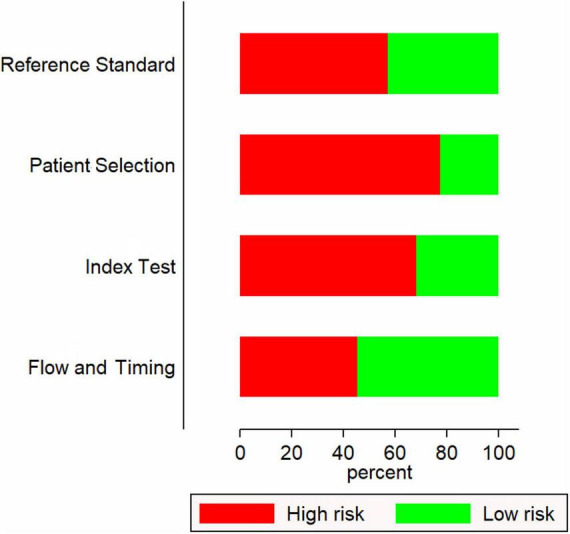
Quality assessment of included studies.

Most of the included studies (17 out of 22) were retrospective in nature. Most were conducted in China (10 studies) followed by Korea (5 studies) and Japan (4 studies). The sample sizes ranged from 48 to 5151. Most studies have used the American Heart Association (AHA) guidelines to diagnose Kawasaki disease followed by Japanese Kawasaki Disease Research Committee (JKDRC) guidelines (Table 1). Fifteen studies had a higher risk of bias (Figure 2).

**TABLE 1:** Characteristics of the included studies (*N* = 22).

Study no	First author and year	Country	Study design	Sample size	Diagnostic criteria for Kawasaki disease	Indicators for predicting resistant Kawasaki disease	Indicator acquisition time	Cut-off point for indicators	Risk of bias
1	Chen et al. (25)	China	Retrospective	92	AHA	NLR	Before IVIG administration	NLR = 2.51	Higher
2	Cho et al. (23)	Korea	Retrospective	196	AHA	NLR	Before IVIG administration	NLR = 5	Higher
3	Faim et al. (38)	Portugal	Retrospective	48	AAP	CRP	Before IVIG administration	CRP = 15.1 mg/dL	Lower
4	Ha et al. (13)	Korea	Retrospective	587	AHA	NLR	Before and after IVIG administration	NLR = 1.26 (after) NLR = 5.49 (before)	Higher
5	Iwashima et al. (24)	Japan	Retrospective	91	JKDRC	CRP	Before IVIG administration	CRP = 8 mg/dL	Higher
6	Kanai et al. (35)	Japan	Retrospective	520	JKDRC	NLR and PLR	Before IVIG administration	NLR = 4.11 PLR = 119	Lower
7	Kawamura et al. (14)	Japan	Retrospective	405	JKDRC	NLR and PLR	Before and after IVIG administration	NLR = 1.27 and PLR = 201 (after) NLR = 3.83 and PLR = 150 (before)	Higher
8	Kim et al. (26)	Korea	Prospective	129	JKDRC	CRP	Before IVIG administration	CRP = 6.88 mg/dL	Lower
9	Kim et al. (22)	Korea	Retrospective	5151	AHA	CRP	Before IVIG administration	CRP = 3.68 mg/dL	Higher
10	Lee et al. (28)	Korea	Retrospective	91	AHA	CRP	Before and after IVIG administration	CRP = 8.3 mg/dL (before) and 3.4 mg/dL (after)	Higher
11	Li et al. (36)	China	Retrospective	957	AHA	CAR	Before IVIG administration	CAR = 3.15	Higher
12	Li et al. (32)	China	Retrospective	1257	AHA	NLR and PLR	Before IVIG administration	NLR = 3.58 PLR = 164	Higher
13	Liu et al. (33)	China	Prospective	542	AHA	NLR and PLR	Before IVIG administration	NLR = 3.37 PLR = 124	Lower
14	Liu et al. (29)	China	Prospective	831	AHA	NLR and PLR	Before IVIG administration	NLR = 3.24 PLR = 134	Lower
15	Liu et al. (30)	China	Prospective	550	AHA	CRP and CAR	Before IVIG administration	CRP = 57.7 mg/L	Lower
16	Lu et al. (27)	China	Retrospective	94	AHA	CRP	Before IVIG administration	CRP = 45 mg/L	Higher
17	Nandi et al. (20)	India	Prospective	72	AHA	CRP	Before IVIG administration	CRP = 40 mg/L	Lower
18	Takeshita et al. (15)	Japan	Retrospective	437	JKDRC	NLR and PLR	Before IVIG administration	NLR = 3.83 PLR = 150	Higher
19	Türkuçar et al. (34)	Turkey	Retrospective	94	JKDRC	NLR and CRP	Before IVIG administration	NLR = 1.69	Higher
20	Wu et al. (21)	China	Retrospective	282	AAP and AHA	NLR and PLR	Before IVIG administration	NLR = 2.69 PLR = 110.92	Higher
21	Xie et al. (31)	China	Retrospective	410	JKDRC	CRP	Before IVIG administration	CRP = 100 mg/L	Higher
22	Yuan et al. (37)	China	Retrospective	404	AHA	NLR and PLR	Before and after IVIG administration	NLR = 4.36 and PLR = 162 (before) NLR = 1.45 and PLR = 196 (after)	Higher

AHA, American Heart Association; AAP, American Academy of Pediatrics; JKDRC, Japanese Kawasaki Disease Research Committee; NLR, Neutrophil Lymphocyte Ratio; PLR, Platelet lymphocyte ratio; CRP, C-reactive protein; CAR, C-reactive protein to albumin ratio; IVIG, Intravenous immunoglobulin.

### Predictive accuracy of neutrophil-to-lymphocyte ratio

**FIGURE 3:**
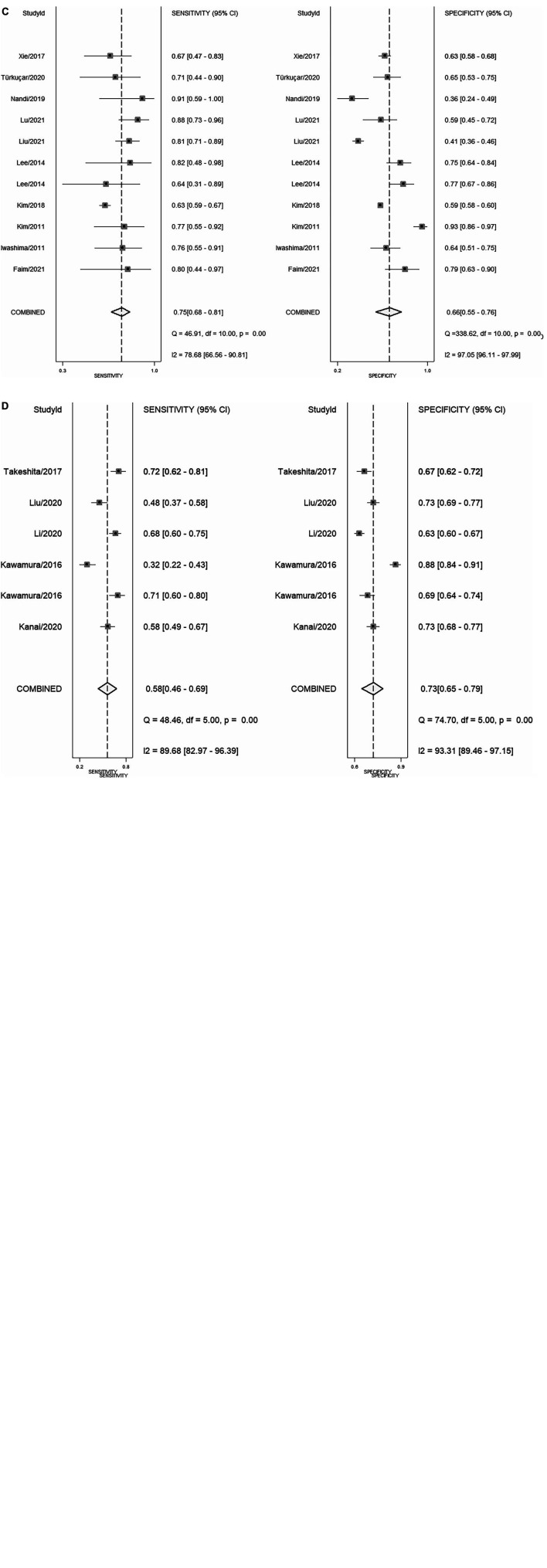
Forest plot **(A)** NLR; **(B)** PLR; **(C)** CRP; **(D)** NLR and PLR combination.

**FIGURE 4:**
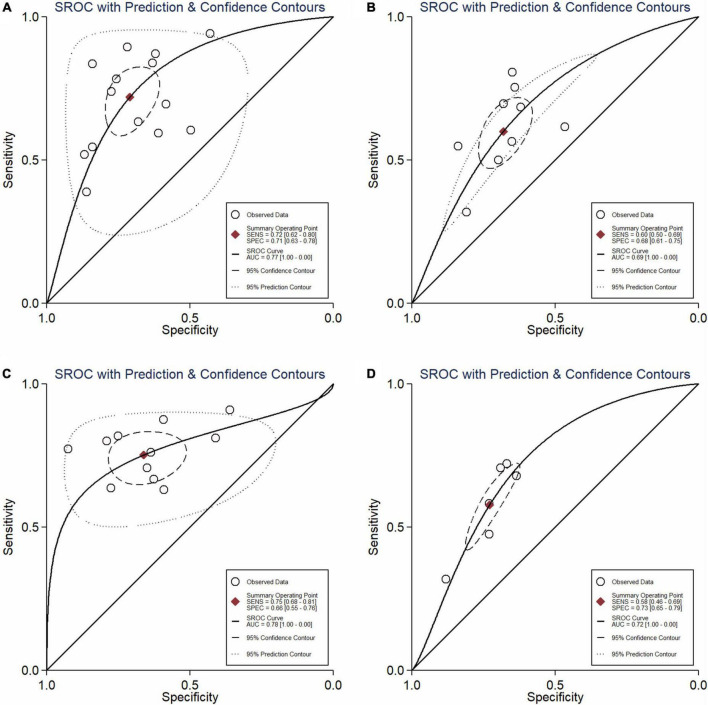
Summary ROC curve **(A)** NLR; **(B)** PLR; **(C)** CRP; **(D)** NLR and PLR combination.

**FIGURE 5:**
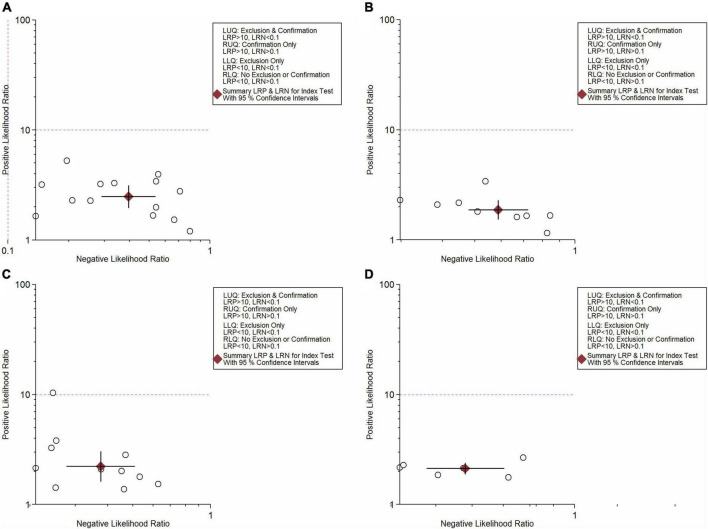
LR scattergram **(A)** NLR; **(B)** PLR; **(C)** CRP; **(D)** NLR and PLR combination.

**FIGURE 6:**
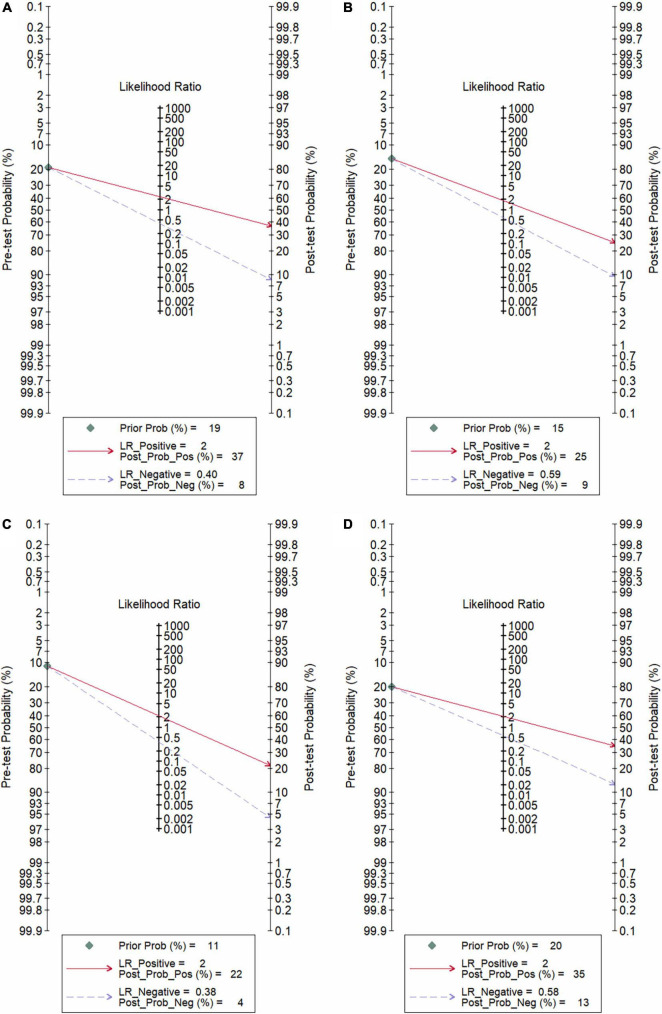
Fagan plot **(A)** NLR; **(B)** PLR; **(C)** CRP; **(D)** NLR and PLR combination.

**FIGURE 7:**
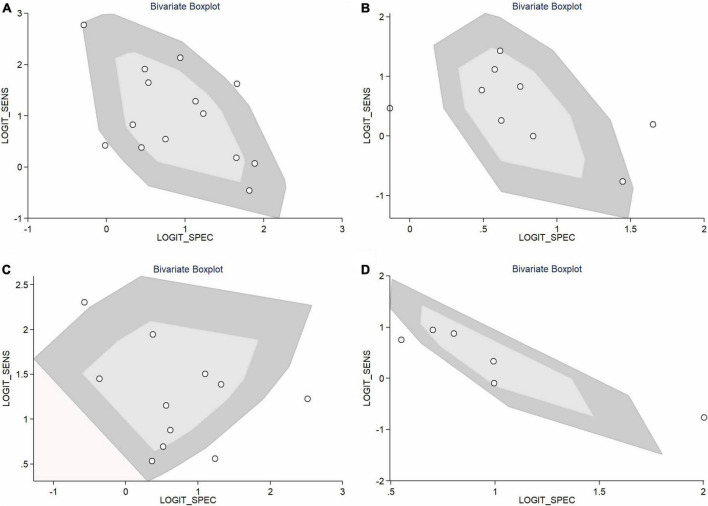
Bivariate box plot **(A)** NLR; **(B)** PLR; **(C)** CRP; **(D)** NLR and PLR combination.

**FIGURE 8:**
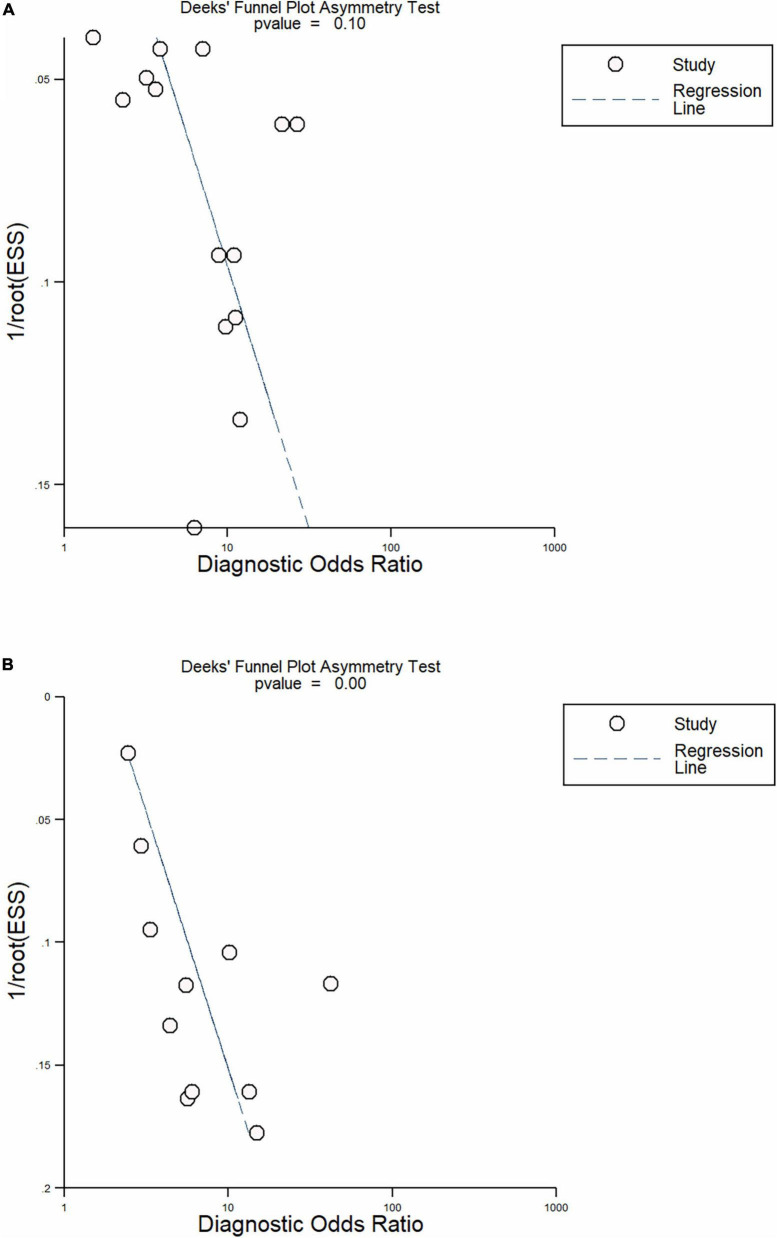
Funnel plot **(A)** NLR; **(B)** CRP.

In total, 14 included studies reported on the utility of NLR for predicting resistant Kawasaki disease. Pooled sensitivity and specificity of NLR for predicting resistant KD were 72% (95% CI: 62%, 80%) and 71% (95% CI: 63%, 78%), respectively, with an AUC value of 0.77 (Figures 3A, 4A). The DOR was 6 (95% CI: 4, 10), LRP was 2.5 (95% CI: 2, 3.1), and LRN was 0.40 (95% CI: 0.29, 0.54). LRP and LRN in the right lower quadrant of the LR scattergram (Figure 5A) suggest no confirmation or exclusion value for NLR. As shown in Figure 6A, NLR has a good clinical value for predicting resistant Kawasaki disease (Positive: 37%; Negative: 8%), compared to the pre-test probability (19%). As shown in Figure 7 and by the bivariate box plot (Figure 7A), there was a significant inter-study heterogeneity (χ^2^ *p* < 0.001, I^2^ > 75%). There was no publication bias, as shown by Deek’s test and the symmetrical funnel plot (Figure 8A).

### Predictive accuracy of platelet-to-lymphocyte ratio

Nine studies reported the use of PLR for predicting resistant Kawasaki disease with pooled sensitivity and specificity of 60% (95% CI: 50%, 69%) and 68% (95% CI: 61%, 75%), respectively, and an AUC value of 0.69 (Figures 3B, 4B). The DOR was 3 (95% CI: 2, 5), LRP was 1.9 (95% CI: 1.5, 2.3), and LRN was 0.59 (95% CI: 0.48, 0.72). Positions of LRP and LRN on the LR scattergram (Figure 5B) indicated no confirmation or exclusion for PLR. PLR has a moderate clinical value in predicting resistant KD (Positive: 25%; Negative: 9%) that differed moderately from the pre-test probability (15%; Figure 6B). There was a significant inter-study variability (χ^2^ *p* < 0.001 and an I^2^ > 75%). This heterogeneity was confirmed by a bivariate box plot (Figure 7B). We could not estimate the publication bias due to the small number of studies.

### Predictive accuracy of C-reactive protein

In total, 11 included studies reported on the utility of CRP for predicting resistant KD, with pooled sensitivity and specificity of 75% (95% CI: 68%, 81%) and 66% (95% CI: 55%, 76%), respectively, with an AUC value of 0.78 (Figures 3C, 4C). The DOR was 6 (95% CI: 3, 10), LRP was 2.2 (95% CI: 1.6, 3.0), and LRN was 0.38 (95% CI: 0.28, 0.51). As shown in Figure 5C, LRP and LRN were in the right lower quadrant of the LR scattergram, which suggests that CRP is not a suitable indicator for confirmation or exclusion. Figure 6C shows a moderate clinical value of CRP for predicting resistant Kawasaki disease (Positive: 22%; Negative: 4%), moderately different compared to the pre-test probability (11%). Significant inter-study heterogeneity with a χ^2^ *p*-value < 0.001 and an I^2^ > 75% was confirmed by bivariate box plot (Figure 7C). Deek’s test and the asymmetrically shaped funnel plot indicated publication bias (*p* < 0.001) (Figure 8B).

### Predictive accuracy of the combination of neutrophil-to-lymphocyte ratio and platelet-to-lymphocyte ratio

Six studies reported the ability of NLR-PLR combination to predict the resistant Kawasaki disease with pooled sensitivity and specificity of 58% (95% CI: 46%, 69%) and 73% (95% CI: 65%, 79%), respectively, and an AUC value of 0.72 (Figures 3D, 4D). The DOR was 4 (95% CI: 3, 5), LRP was 2.1 (95% CI: 1.9, 2.4), and LRN was 0.58 (95% CI: 0.48, 0.70). LR scattergram (Figure 5D) showed that the NLR-PLR combination is not suitable for confirmation or exclusion. Figure 6D shows a good clinical utility of NLR and PLR combination for predicting IVIG-resistant KD (Positive: 35%; Negative: 13%) that differed significantly from the pre-test probability (20%). There was a significant inter-study heterogeneity (χ^2^ *p* < 0.001; I^2^ > 75%), shown also by the bivariate box plot (Figure 7D). Publication bias was not assessed due to a small number of studies.

### Predictive accuracy of PAR

Only two of the included studies reported on the utility of CAR for predicting resistant Kawasaki disease. It was not possible, therefore, to pool the results and obtain the sensitivity, specificity, and other predictive accuracy indices. However, both studies have reported moderate predictive accuracy of sensitivity that ranged from 61 to 70% and specificity of 55–75%.

## Discussion

Our systematic literature search found 22 studies that reported the use of NLR, PLR, CRP, CAR, and NLR in combination with PLR to predict resistant KD. Out of these markers, CRP showed the highest pooled sensitivity (75%) followed by NLR (72%). However, the pooled specificity was higher for NLR + PLR (73%) followed by NLR alone (71%) for the prediction of resistant KD. CRP and NLR had better overall sensitivity and specificity compared to all other markers. CRP is a protein that is produced by the hepatocytes in response to inflammation. CRP levels rise during the acute phase of the inflammatory process after the IL-6 and TNF-alpha activation (cytokine activation) (39), making it a well-established marker for Kawasaki disease (10, 40, 41). The extent of the increase in the levels of CRP can in turn provide evidence of the possible resistance KD.

Our results, showing the high predictive value of NLR, are in line with the previous review assessing the NLR accuracy for predicting the resistant KD (42). Neutrophils reflect the ongoing inflammatory process, while the lymphocytes act as a marker for immune regulatory response (25). Therefore, NLR can be a marker of balance between the inflammatory process and the immune regulatory response (43). The clinical value of NLR in our study was further confirmed by Fagan’s nomogram, which showed a marked increase in the post-NLR probability vs. pre-NLR probability.

In our review, PLR was identified as a moderately useful marker for predicting the resistant Kawasaki disease. Like the other inflammatory markers, elevated platelet counts are indicative of systemic infection and inflammation, since the increased levels of the proinflammatory cytokines cause the proliferation of megacaryocytes (44, 45). The combination of PLR and NLR showed good specificity but a weaker sensitivity, thus making it not useful as a marker for the identification of resistant KD.

Not enough studies, included in our review, evaluated CAR as a prognostic marker. Therefore, it was not possible to pool the evidence and obtain pooled estimates. However, studies reporting the accuracy of CAR for the identification of resistant KD, reported that its sensitivity and specificity were moderate. CAR, therefore, can be considered a useful marker.

Based on our review findings, all analyzed markers had moderate to a high level of sensitivity and specificity. We could not identify any specific index as the preferred marker for predicting resistant KD. This makes it a very useful marker for the prediction. Further studies, focusing on building a scoring system that combines all the important markers that can be applied to predict the resistant Kawasaki disease at the early stages, are needed.

Our review has certain strengths. This meta-analysis involved 22 studies with a large sample size of > 7000 participants. We comprehensively assessed multiple inflammatory markers and identified the level of predictive accuracy for resistant Kawasaki disease. However, our review has certain limitations. Each study included in the review had wide range of biomarkers value considered to put the patient at high risk of IVIG failure. It is unclear which threshold is associated with such risk. Stratification of results based on cut-off was also not possible given the wide range and difference across the studies. Studies included in our review were of lower quality across most of the domains with significant publication bias for most markers, which might affect the credibility of our analysis. Moreover, inter-study heterogeneity that was detected may limit our ability to interpret the pooled results.

In conclusion, our study may contribute to further improvement of the clinical management of resistant Kawasaki disease. Our results suggest that inflammatory markers such as NLR, CRP, and PLR can be utilized as a useful screening and predictive tool. Moreover, early prediction of IVIG resistance in KD patients may lead to early initiation of additional anti-inflammatory therapy and more effective therapeutic management. There is a need for further updated reviews that would compare the predictive performance of the various individual and combined inflammatory markers in KD patients.

## Data availability statement

The original contributions presented in this study are included in the article/supplementary material, further inquiries can be directed to the corresponding author.

## Author contributions

CL and JW conceived and designed the study, collected the data, and performed the analysis. JW was involved in the writing of the manuscript and is responsible for the integrity of the study. Both authors have read and approved the final manuscript.
